# Dietary Patterns Based on Estimated Glomerular Filtration Rate and Kidney Function Decline in the General Population: The Lifelines Cohort Study

**DOI:** 10.3390/nu12041099

**Published:** 2020-04-16

**Authors:** Qingqing Cai, Louise H. Dekker, Stephan J. L. Bakker, Martin H. de Borst, Gerjan J. Navis

**Affiliations:** 1 Department of Medicine, Division of Nephrology, University Medical Center Groningen, University of Groningen, Hanzeplein 1, 9713 GZ Groningen, The Netherlands; l.h.dekker@umcg.nl (L.H.D.); s.j.l.bakker@umcg.nl (S.J.L.B.); m.h.de.borst@umcg.nl (M.H.d.B.); g.j.navis@umcg.nl (G.J.N.); 2 Aletta Jacobs School of Public Health, 9747 AD Groningen, The Netherlands

**Keywords:** chronic kidney disease, eGFR decline, reduced rank regression, dietary pattern, Mediterranean diet

## Abstract

No specific dietary patterns have been established that are linked with loss of kidney function. We aimed to identify an estimated glomerular filtration rate-based dietary pattern (eGFR-DP) and to evaluate its association with eGFR decline and chronic kidney disease (CKD) incidence in the general population. We included 78,335 participants from the Lifelines cohort in the Northern Netherlands. All participants had an eGFR >60 mL/min/1.73 m^2^ at baseline and completed a second visit five years later. The eGFR-DP was constructed at baseline using a 110-item food frequency questionnaire by reduced rank regression, stratified by sex. Logistic regression was performed to evaluated the association between the eGFR-DP score and either a ≥20% eGFR decline or incident CKD. Among women, eGFR-DP were characterized by high consumption of egg, cheese, and legumes and low consumption of sweets, white meat, and commercially prepared dishes. In men, eGFR-DP were characterized by high consumption of cheese, bread, milk, fruits, vegetables, and beer and low consumption of white and red meat. A higher eGFR-DP score was associated with a lower risk of a ≥20% eGFR decline (OR 4th vs. 1st quartile, women: 0.79 [95% CI: 0.73–0.87]; men: 0.67 [0.59–0.76]). The association between the eGFR-DP score and CKD incidence was lost upon adjustment for baseline eGFR. Our results provide support for dietary interventions to prevent kidney function decline in the general population.

## 1. Introduction

Diet is a modifiable factor that plays an important role in accelerated kidney function decline and incident chronic kidney disease (CKD) [1]. Previous studies predominantly focused on the effects of single nutrients, foods, or food groups in this context [2,3,4]. However, a dietary pattern-based approach may be more informative, as it provides a broader picture of food and nutrient intake [5]. Although some individual studies have shown that the Mediterranean diet (higher intake of fruit, vegetables, legumes, cereals, and fish) or the Dietary Approach to Stop Hypertension (DASH) diet (higher intake of fruits, vegetables, and whole grain) have been associated with a lower risk of estimated glomerular filtration rate (eGFR) decline [6,7], a recent meta-analysis did not confirm associations of these dietary patterns with eGFR decline overall [8]. These findings raise questions about whether kidney-specific healthy dietary patterns exist, whether their composition differs from more general healthy dietary patterns, and whether these are consistently associated with eGFR decline and CKD incidence.

Reduced rank regression (RRR) is a method that derives dietary patterns in an exploratory way while using a priori knowledge on selected response variables (e.g., biomarkers) that are known to be associated with a disease of interest [9,10]. To date, RRR has been applied to derive dietary patterns in several chronic diseases including diabetes [11,12], cardiovascular disease [13], and obesity [14]. In the setting of CKD, eGFR as a key index of renal function might be an ideal response variable to generate a dietary pattern explaining maximum variation in eGFR. Therefore, the RRR method may be particularly useful for the identification of kidney-specific dietary patterns, as these may be strongly linked with kidney outcomes such as incident CKD and eGFR decline in the general population. The identification of kidney-specific dietary patterns may open novel avenues for dietary interventions to prevent CKD.

Therefore, we aimed to identify specific dietary patterns explaining the maximum variation in eGFR using RRR. Subsequently, we evaluated whether the eGFR-based dietary pattern (eGFR-DP) is associated with a ≥20% eGFR decline or incident CKD in a large population-based cohort.

## 2. Materials and Methods

### 2.1. Study Population

The Lifelines cohort study is a population-based cohort to study the health and health-related behaviors in the north of the Netherlands. More than 165,000 participants were recruited between 2006–2013 through invitation by their general practitioners in the three Northern provinces of the Netherlands. From 2014 to 2018, all the participants were invited again to the second assessment. Detailed information about the Lifelines cohort study can be found elsewhere [15]. A total of 78,335 participants were included in the present study. Figure S1 shows flow diagrams outlining the study population, exclusions, and missing data.

All participants signed informed consent before entering the cohort, and the research protocol and data access application were reviewed and approved by the medical ethical review committee of the University Medical Center Groningen.

### 2.2. Dietary Intake Assessment

Dietary intake was evaluated with a self-administered 110-item food frequency questionnaire (FFQ) [15]. The FFQ used in Lifelines study has been validated previously [16,17]; it included questions on the frequency and portion size of 110 food items during the last four weeks. To correct for the potential under- or over-reporting on the dietary questionnaire, participants with unreliable energy intake were excluded from the analysis. Energy intake/basal metabolic rate (EI/BMR) was used to evaluate the reliability of dietary intake. EI/BMR <0.5 and >2.75 were considered unreliable; EI/BMR between 0.5 and 2.75 were considered reliable [18,19]. In the present study, food items were manually classified into 50 food groups based on similarities in food and nutrient composition, in line with recent studies [20,21] (Table S1).

### 2.3. Other Variables

Sociodemographic characteristics and health-related behavior data were assessed based on self-administered questionnaires. Education level was classified into four groups (low: never been to school, elementary school only, or lower vocational or secondary school; middle: intermediate vocational school or intermediate/higher secondary school; high, higher vocational school or university; unknown or no answer). Income level was defined as a mean gross monthly income of: low: <1000 euro; middle: 1000–3000 euro; high: >3000 euro; unknown or no answer. Smokers referred to current smokers. The time spent on moderate to vigorous physical activity was evaluated by the validated Short Questionnaire to Assess Health-enhancing physical activity (SQUASH) questionnaire. Body mass index (BMI) was calculated as weight (kg) divided by height squared (m^2^). Body surface area (BSA) was calculated by the Du Bois formula: $BSA=0.007184\times Weight^{0.425}\times Height^{0.725}$. Biochemistry and renal function assessment included blood laboratory assessment and urine laboratory assessments, of which detailed information has been published previously [22]. eGFR was calculated using the creatinine-based Chronic Kidney Disease Epidemiology Collaboration equation (CKD-EPI) [23]. CKD was defined as an eGFR < 60 mL/min/1.73 m^2^. Participants were categorized as having diabetes if they had self-reported diabetes and/or a non-fasting plasma glucose ≥ 11 mmol/L and/or a measured glycated hemoglobin (HbA1c) ≥ 6.5% and/or use of oral anti-diabetics and/or insulin. Cardiovascular disease included self-reported coronary artery disease, heart failure and/or stroke. Hypertension was defined as blood pressure > 140/90 mmHg or use of anti-hypertensive medication.

### 2.4. Prospective Outcomes

The primary outcome was a ≥20% eGFR decline between the first and second assessments. The secondary outcome of this study was CKD incidence, defined as the development of an eGFR < 60 mL/min/1.73 m^2^ at the second assessment in participants who were free of CKD at the first assessment.

### 2.5. Dietary Pattern Analysis

RRR was used to derive dietary patterns predictive of eGFR decline and CKD incidence. Detailed information about RRR, including SAS code and its application in nutritional epidemiology has been described by Hoffmann et al. [10]. Briefly, RRR identifies linear functions of predictor variables (food groups) that explain maximized variation in the response variables (biomarkers). To perform RRR, we first distinguished two types of observed variables: predictor variables (50 food groups) and response variable (baseline eGFR). eGFR is a biomarker of renal function and it is estimated from equations using serum creatinine, age race, sex, and body size [23]. The majority of the population in this cohort are Caucasians, therefore, we used age- and BSA-adjusted eGFR as a response variable and performed RRR separately by sex. The dietary pattern score was calculated as the sum of z-standardized consumptions (mean = 0, standard deviation = 1) of 50 food groups multiplied by an individual weight (factor loading). Food groups with high absolute factor loading ≥ 0.2 were considered to be the main contributors to the score. In order to decrease the data dependency of the pattern variables, a simplified dietary pattern score was constructed by summing the unweighted standardized food variables (g/day) with high absolute factor loadings (≥0.2) while retaining the direction of the factor loading [10,24]. Then, this eGFR-DP score was divided into quartiles.

The Mediterranean Diet Score (MDS) in this study was calculated as a nine-point MDS according to Trichopoulou et al. [25]. Briefly, nine food groups were included in MDS: vegetables, legumes, cereal, fruit and nuts, and fish, as beneficial components; meats, poultry, and dairy products, as detrimental components; and alcohol intake. Participants received a score 1 for each of the beneficial components if their intake was above the sex-specific median in grams per day; additionally, an intake below the median for detrimental components received a score of 1. For alcohol, a value of 1 was given to men who consumed between 10 and 50 g/day or to women who consumed between 5 to and 25 g/day. The MDS varies between 0 and 9. Three groups were created according to the tertiles of the MDS.

### 2.6. Statistical Analysis

Baseline characteristics are presented according to the quartiles of the eGFR-DP score for women and men. Multivariable logistic regression was applied to evaluate the association between the eGFR-DP score and a ≥20% eGFR decline or CKD incidence, adjusted for potential confounders. Odds ratios (OR) and 95% confidence intervals (CI) were calculated across the quartiles of the eGFR-DP score. In logistic regression, initially, we adjusted for age and BSA (Model 1). Then, we adjusted for clinical variables including BMI, waist circumference, cholesterol, triglycerides, and present diabetes, hypertension, or cardiovascular disease in Model 1 (Model 2). After that, we additionally adjusted for health-related behavior variables, including physical activity, smoker, and total energy intake (Model 3). Furthermore, socioeconomic characteristics were added to model 3 (Model 4). Finally, we additionally adjusted for baseline eGFR (Model 5). We used splines to visualize the association of eGFR-DP score with a ≥20% eGFR decline by fitting logistic regression models according to Model 5. The same models were used to study the association between the MDS and both outcomes.

*P* values for trend were computed in the study. A two-tailed *P* value < 0.05 was considered statistically significant. The statistical analyses were conducted using R version 3.4.2 (Vienna, Austria) and SAS software (version 9.4; SAS Institute, Cary, NC).

## 3. Results

Among the 78,335 participants in this study, the mean (±standard deviation) age of the participants was 46 ± 13 years (range 18–90 years). Fifty-eight percent of the participants were women. The baseline eGFR was 95.4 ± 15.4 mL/min/1.73 m^2^ in women and 96.7 ± 14.2 mL/min/1.73 m^2^ in men.

### 3.1. eGFR-Based Dietary Pattern (eGFR-DP)

RRR was applied to identify the sex-stratified eGFR-DP. Initially, all 50 food groups were ranked by decreasing absolute factor loadings in men and women (Tables S2 and S3). The derived pattern scores explained 1.48% and 2.33% of the variation in eGFR, and explained 2.37% and 2.61% in food groups among women and men, respectively.

Ten food groups with an absolute factor loading ≥ 0.2 were considered to be the main contributors to the women and men dietary patterns. With increasing quartiles of the eGFR-DP score, the quality of the food intake seemed to be better, with high consumption of eggs, low-fat cheese, high-fat cheese, and legumes and low consumption of sweetened dairy drinks, desserts, cake and cookies, sweet sandwich toppings, white meat, and commercially prepared dishes in women. The identified dietary pattern in men was characterized by high consumption of high-fat cheese, bread, full-fat milk, fruits, vegetables, beer, low-fat cheese, and legumes and low consumption of white meat and red meat. The 10 food groups together explained 66% and 76% of food pattern score variation in women and men, respectively. Table 1 and Table 2 show the factor loading and the median intake of the 10 most important food groups across quartiles of the eGFR-DP score in women and men.

### 3.2. Baseline Characteristics across the Quartiles of eGFR-DP Score

Baseline characteristics across the quartiles of the eGFR-DP score in women and men are shown in Tables S4 and S5. At baseline, higher eGFR-DP scores were associated with older age and slightly lower baseline eGFR both in women and men. Furthermore, the proportions of diabetes, hypertension, and cardiovascular disease were higher in the highest compared to the lowest quartile (*P*-trend < 0.001), both in women and men. Interestingly, with an increasing eGFR-DP score, the proportion of protein consumption was higher, carbohydrate consumption was lower, and fat consumption was higher in women, while the opposite was true for all three parameters in men. In the highest quartile, the proportion with low education was higher, while the proportion of low income was lower compared to the lowest quartile both in women and men.

### 3.3. eGFR-DP Scores and Renal Outcomes

**Figure 1:**
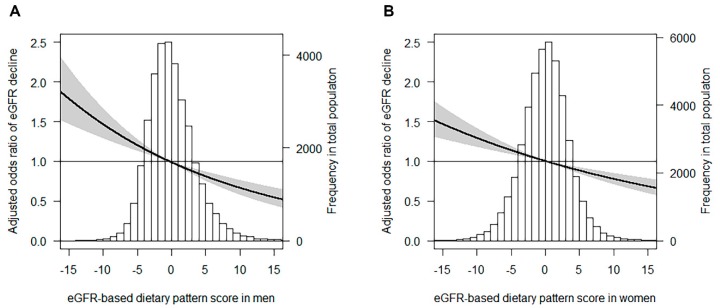
Adjusted odds ratio of a ≥20% eGFR decline in men and women. Data were fit by a multivariable logistic regression using the median value of eGFR-based dietary pattern score as the reference value (odds ratio = 1). The black line represents the adjusted odds ratio and the grey area represents the 95% confidence interval. Prevalent odds ratios of a ≥20% eGFR decline in men (**A**) or women (**B**) are shown, adjusted for age, BSA, BMI, waist circumference, cholesterol, triglycerides, diabetes, hypertension, cardiovascular disease, physical activity, smoker, total energy intake, education and income, and baseline eGFR.

At the follow-up assessment, 7610 (9.7%) participants had a ≥20% eGFR decline and 2072 (2.6%) individuals developed CKD. Individuals in the highest quartile of the eGFR-DP score had a lower risk of a ≥20% eGFR decline compared with the lowest quartile (women: OR 0.79 [95% CI: 0.73–0.87], men OR 0.67 [0.59–0.76]), independent of other potential risk factors (Table 3, Model 5). The association between the continuous eGFR-DP score and a ≥20% eGFR decline in men and women using multivariable logistic regression analysis are presented in Figure 1A,B. Individuals in the highest quartile also had a lower risk of incident CKD compared with the lowest quartile (Table 4, Models 1–4), but the associations were no longer significant upon adjustment for baseline eGFR.

### 3.4. MDS and Renal Outcomes

We also studied the associations of the MDS with a ≥20% eGFR decline or incident CKD (Tables S6 and S7). Interestingly, a higher MDS was only weakly associated with a lower risk of ≥20% eGFR decline in men, while no significant associations were found in women for both outcomes.

## 4. Discussion

In the present study, we identified a sex-specific eGFR-DP, and subsequently showed that this pattern was inversely associated with a ≥20% eGFR decline in the population-based Lifelines cohort. The eGFR-DP in women was characterized by high consumption of egg, cheese, and legumes and low consumption of sweets, white meat, and commercially prepared dishes. In men, the eGFR-DP was characterized by high consumption of cheese, bread, milk, fruits, vegetables, and beer and low consumption of white meat and red meat. To our knowledge, this is the first study applying RRR to identify an eGFR-DP in the general population. Although the derived dietary patterns partly overlapped for men and women but also show differences, it could be useful to consider different advice for men and women to prevent CKD progression. Importantly, compared to the MDS, the eGFR-DP had a stronger association with eGFR decline, supporting the concept that RRR is a useful tool to identify kidney-specific dietary patterns. The association between the eGFR-DP and incident CKD (defined as an eGFR < 60 mL/min/1.73 m^2^ during follow-up) lost significance upon adjustment for baseline eGFR, possibly since eGFR in itself is a very strong risk factor for incident CKD.

Dietary predictors of renal function decline and incident CKD have been investigated at different levels including nutrients, foods, and dietary patterns. Rebholz et al. found that higher intake of plant protein, magnesium, calcium, and phosphorus were inversely associated with incident CKD [26]. Some individual studies showed that the Mediterranean diet [7] and DASH diet [6,27] were associated with a lower risk of CKD development and progression. Lin et al. identified dietary patterns using principal component analysis (PCA), and concluded that a Western diet was associated with rapid eGFR decline while a DASH diet was associated with slower eGFR decline in older white women [6]. Of note, a meta-analysis of cohort studies suggested that a healthy dietary pattern rich in fruits and vegetables, fish, legumes, whole grain, and fiber intake and low in red meat, sodium, and refined sugar intake was associated with a lower risk of incident CKD and albuminuria, but not with eGFR decline [8]. In the present study, we could identify sex-specific eGFR-DPs that, in contrast with the MDS in our cohort, were independently associated with kidney function decline. The components of our eGFR-DP partly overlap with other healthy diets, i.e., a high intake of vegetables, fruits, and legumes and low intake of red meat and sweets. The role of dairy products in relation to incident CKD and eGFR decline is controversial. In our study, higher dairy intake was associated with a higher eGFR in both sexes, whereas it is considered a detrimental component in the MDS.

Ample evidence suggested that high protein intake is a risk factor for the progression of established CKD [28,29]. However, other studies reported no deleterious effects of long-term high-protein consumption on kidney function in the general population [30,31]. A recent study even found that in renal transplant recipients, high protein intake is associated with a lower risk of graft failure and mortality [32]. The eGFR-DP identified in the present study suggests that the effect of dietary protein intake on renal function seems to depend not only on the specific population and protein quantity, but also on the protein source. Some studies found that red meat protein intake over years increased the risk of albuminuria and eGFR decline, while white meat and dairy proteins had no such effect, and fruit and vegetable protein had a protective effect [6,33]. Growing evidence indicates that the protein source plays an important role in the prevention and management of CKD, suggesting that a shift from animal to plant source of protein might be beneficial [34,35], an assumption supported by our current results. One of the main theories in nephrology holds that high-protein consumption increases renal blood flow, glomerular pressure, and glomerular filtration rate acutely, but results in glomerular sclerosis and loss of renal function on the long term [36,37]. In our study, however, participants in the highest eGFR-DP quartile who consumed more protein-rich food did not demonstrate a more rapid decline in eGFR. Therefore, in this general population cohort, the subtle differences in baseline eGFR linked to higher protein intake apparently do not reflect “hyperfiltration” as a factor driving subsequent renal function loss.

The major strengths of this study include its large sample size and the application RRR to identify eGFR-based dietary patterns in the general population. In addition, some limitations should be noted. First, the FFQ was self-administrated, thus, a high proportion of participants excluded due to unreliable dietary data. Second, we could not include albuminuria in the definition of CKD due to unavailable data at the second assessment, which may underestimate the true CKD incidence. Third, the eGFR-DP explained only a small percentage of the variation in eGFR at baseline, and the average eGFR was normal in this population-based cohort. Therefore, our findings should be interpreted with caution. Finally, our study was conducted in The Netherlands, with dietary assessment validated for The Netherlands. This precludes generalization to other areas, as dietary habits and patterns are different across the world. For future studies, RRR is exquisitely suited to dissect the role of particular nutrients (such as protein content and protein source) within the context of cultural differences in dietary patterns. Future studies are needed to confirm our observational data, and prospective studies might be done to establish whether dietary modifications in accordance with the dietary patterns we found may lead to the preservation of renal function.

In conclusion, an eGFR-based dietary pattern characterized by a high intake of egg, dairy products, fruits, vegetables, and legumes and low intake of meat and sweets was independently associated with a lower risk of eGFR decline in the general population of the Northern Netherlands. Our study provides novel insights into kidney-specific dietary patterns that are associated with changes in renal function in the general population, and it may open novel avenues for dietary interventions to prevent renal function decline and CKD.

## Figures and Tables

**Table 1:** Food groups strongly associated with the estimated glomerular filtration rate (eGFR)-based dietary pattern score in women obtained by reduced rank regression.

		Quartiles of Dietary Pattern Score in Women ^2^ (*n* = 45,746) (g/Day)				
Food Groups	Factor Loading ^1^	1	2	3	4	P-*Trend*
**High intake**						
Eggs	0.42	7.2 (4.5–14.3)	7.2 (4.5–17.9)	7.2 (4.5–17.9)	17.9 (7.2–32.2)	<0.001
Low-fat cheese	0.23	0 (0–4.6)	0.4 (0–8.2)	2.0 (0–11.8)	5.1 (0–17.9)	<0.001
High-fat cheese	0.23	11.9 (4.6–22.4)	14.3 (5.9–26.1)	16.3 (6.5–30.5)	22.5 (8.5–42.8)	<0.001
Legumes	0.20	0 (0–6.6)	4.4 (0–11.0)	4.4 (0–11.0)	11.0 (0–17.6)	<0.001
**Low intake**						
Sweetened dairy drinks	−0.27	101.6 (53.6–174.6)	80.3 (40.4–139.4)	58.8 (23.4–104.8)	40.1 (8.9–83.9)	<0.001
Desserts	−0.26	13.4 (3.4–39.9)	8.3 (0–21.0)	3.4 (0–8.3)	3.4 (0–8.3)	<0.001
Cakes and cookies	−0.24	46.3 (30.4–66.4)	35.3 (23.1–49.9)	27.8 (17.4–40.8)	20.4 (11.6–33.5)	<0.001
Sweet sandwich toppings	−0.22	19.3 (9.7–28.1)	10.8 (2.7–19.4)	5.4 (1.3–14.0)	2.2 (0–9.7)	<0.001
White meat	−0.22	12.4 (8.4–19.4)	10.8 (6.8–15.6)	9.4 (5.4–13.5)	7.4 (2.7–11.2)	<0.001
Commercially prepared dishes	−0.21	33.3 (14.4–53.8)	31.3 (11.8–48.6)	21.2 (5.9–35.6)	13.2 (0–32.4)	<0.001

Data are shown with median and interquartile range (25%–75%). ^1^ Factor loading was obtained directly by reduced rank regression. ^2^ Dietary pattern score was the sum of the unweighted standardized food variables with high factor loadings (≥0.2).

**Table 2:** Food groups strongly associated with the eGFR-based dietary pattern score in men obtained by reduced rank regression.

		Quartiles of Dietary Pattern Score in Men ^2^ (*n* = 32,589) (g/Day)				
Food Groups	Factor Loading ^1^	1	2	3	4	P–Trend
**High intake**						
High–fat cheese	0.38	11.6 (3.6–22.9)	16.6 (6.3–30.6)	21.2 (7.8–39.7)	28.3 (10.2–55.5)	<0.001
Bread	0.34	129.9 (90.2–165.6)	150.1 (115.0–197.0)	169.9 (132.8–211.1)	198.0 (148.0–257.7)	<0.001
Full–fat milk	0.23	0 (0–11.9)	0 (0–34.6)	5.4 (0–71.8)	38.3 (0–139.4)	<0.001
Fruits	0.23	42.3 (16.9–110.1)	84.6 (42.3–152.4)	110.1 (52.7–220.2)	220.2 (84.6–228.6)	<0.001
Vegetables	0.21	74.3 (41.6–110.5)	81.8 (62.1–113.1)	110.2 (63.5–149.1)	113.1 (76.3–162.5)	<0.001
Beer	0.21	43.0 (0–107.4)	57.3 (11.9–142.8)	71.4 (18.9–171.9)	73.9 (19.1–214.2)	<0.001
Low–fat cheese	0.20	0 (0–3.1)	0 (0–6.9)	0 (0–10.3)	0 (0–17.9)	<0.001
Legumes	0.20	0 (0–11.0)	5.5 (0–16.4)	8.9 (0–17.6)	16.4 (4.4–27.4)	<0.001
**Low intake**						
White meat	−0.33	13.2 (9.5–19.4)	9.6 (6.7–13.9)	8.4 (5.3–12.4)	7.5 (2.3–11.2)	<0.001
Red meat	−0.22	29.4 (20.4–39.5)	24.5 (15.1–32.1)	22.3 (12.9–30.5)	18.5 (9.3–28.2)	<0.001

Data are shown with median and interquartile range (25%–75%). ^1^ Factor loading was obtained directly by reduced rank regression. ^2^ Dietary pattern score was the sum of the unweighted standardized food variables with high factor loadings (≥0.2).

**Table 3:** Risk of ≥20% eGFR decline according to baseline eGFR-based dietary pattern score.

**Women**	**Quartiles of Dietary Pattern Score OR (95% CI)**						**Continuous Dietary Pattern Score**
	**1**	**2**	**3**	**4**	**P for Trend**	**OR (95% CI)**	**P**
Cases/population	1316/11,438	1286/11,436	1215/11,436	1155/11,436		4972/45,746	
eGFR decline ≥20% (%)	11.5	11.2	10.6	10.1	<0.001	10.9	
Model 1	1.00	0.97 (0.90–1.06)	0.91 (0.84–0.99)	0.86 (0.79–0.94)	<0.001	0.93 (0.90–0.97)	<0.001
Model 2	1.00	0.97 (0.90–1.06)	0.91 (0.84–0.99)	0.86 (0.79–0.94)	<0.001	0.93 (0.90–0.97)	<0.001
Model 3	1.00	0.95 (0.88–1.04)	0.88 (0.81–0.96)	0.83 (0.76–0.91)	<0.001	0.92 (0.88–0.95)	<0.001
Model 4	1.00	0.95 (0.88–1.03)	0.88 (0.81–0.96)	0.83 (0.76–0.91)	<0.001	0.92 (0.88–0.95)	<0.001
Model 5	1.00	0.93 (0.86–1.02)	0.86 (0.79–0.94)	0.79 (0.73–0.87)	<0.001	0.90 (0.86–0.93)	<0.001
**Men**	**Quartiles of Dietary Pattern Score OR (95% CI)**						**Continuous Dietary Pattern Score**
	**1**	**2**	**3**	**4**	**P for Trend**	**OR (95% CI)**	**P**
Cases/population	756/8147	660/8148	648/8147	574/8147		2638/32,589	
eGFR decline ≥20% (%)	9.3	8.1	8.0	7.0	<0.001	8.1	
Model 1	1.00	0.85 (0.76–0.94)	0.82 (0.73–0.92)	0.71 (0.64–0.80)	<0.001	0.87 (0.83–0.92)	<0.001
Model 2	1.00	0.85 (0.76–0.95)	0.81 (0.73–0.91)	0.70 (0.63–0.79)	<0.001	0.86 (0.82–0.91)	<0.001
Model 3	1.00	0.84 (0.75–0.94)	0.80 (0.72–0.90)	0.68 (0.60–0.77)	<0.001	0.85 (0.80–0.90)	<0.001
Model 4	1.00	0.84 (0.76–0.94)	0.80 (0.72–0.90)	0.68 (0.60–0.77)	<0.001	0.85 (0.80–0.90)	<0.001
Model 5	1.00	0.84 (0.75–0.94)	0.80 (0.71–0.89)	0.67 (0.59–0.76)	<0.001	0.85 (0.80–0.90)	<0.001

Model 1. Adjusted for age and body surface area (BSA); Model 2. Model 1 plus body mass index (BMI), waist circumference, cholesterol, triglycerides, diabetes, hypertension, and cardiovascular disease; Model 3. Model 2 plus physical activity, smoker, and total energy intake; Model 4. Model 3 plus education and income; Model 5. Model 4 plus baseline eGFR.

**Table 4:** Risk of CKD incidence according to baseline eGFR-based dietary pattern score.

**Women**	**Quartiles of Dietary Pattern Score**				**Continuous Dietary Pattern Score**		
	**1**	**2**	**3**	**4**	**P for Trend**	**OR (95% CI)**	**P**
Cases/population	255/11,438	332/11,436	331/11,436	344/11,436		1262/45,746	
CKD incidence (%)	2.2	2.9	2.9	3.0	0.001	2.8	
Model 1	1.00	0.94 (0.79–1.12)	0.78 (0.66–0.93)	0.67 (0.57–0.80)	<0.001	0.80 (0.74–0.86)	<0.001
Model 2	1.00	0.94 (0.79–1.11)	0.77 (0.65–0.92)	0.67 (0.56–0.79)	<0.001	0.80 (0.74–0.86)	<0.001
Model 3	1.00	0.92 (0.77–1.09)	0.74 (0.62–0.89)	0.64 (0.54–0.77)	<0.001	0.78 (0.72–0.85)	<0.001
Model 4	1.00	0.92 (0.77–1.09)	0.74 (0.62–0.89)	0.64 (0.54–0.77)	<0.001	0.78 (0.72–0.85)	<0.001
Model 5	1.00	1.04 (0.85–1.27)	0.88 (0.72–1.07))	0.88 (0.72–1.08)	0.079	0.93 (0.85–1.01)	0.095
**Men**	**Quartiles of Dietary Pattern Score**					**Continuous Dietary Pattern Score**	
	**1**	**2**	**3**	**4**	**P for Trend**	**OR (95% CI)**	**P**
Cases/population	216/8147	216/8148	195/8147	183/8147		810/32,589	
CKD incidence (%)	2.7	2.7	2.4	2.2	0.056	2.5	
Model 1	1.00	0.77 (0.62–0.94)	0.57 (0.46–0.70)	0.50 (0.40–0.61)	<0.001	0.70 (0.64–0.78)	<0.001
Model 2	1.00	0.78 (0.64–0.96)	0.58 (0.47–0.71)	0.51 (0.41–0.63)	<0.001	0.71 (0.64–0.79)	<0.001
Model 3	1.00	0.80 (0.65–0.98)	0.60 (0.49–0.75)	0.55 (0.44–0.69)	<0.001	0.73 (0.66–0.82)	<0.001
Model 4	1.00	0.80 (0.65–0.98)	0.60 (0.49–0.75)	0.54 (0.43–0.68)	<0.001	0.73 (0.66–0.82)	<0.001
Model 5	1.00	0.90 (0.71–1.14)	0.76 (0.59–0.97)	0.95 (0.73–1.23)	0.372	0.96 (0.85–1.09)	0.578

Model 1. Adjusted for age and BSA; Model 2. Model 1 plus BMI, waist circumference, cholesterol, triglycerides, diabetes, hypertension and cardiovascular disease; Model 3. Model 2 plus physical activity, smoker, and total energy intake; Model 4. Model 3 plus education and income; Model 5. Model 4 plus baseline eGFR.

## Supplementary Materials

The following are available online at https://www.mdpi.com/2072-6643/12/4/1099/s1. Table S1. Classification of FFQ items in the 50 established food groups in Lifelines cohort. Table S2. Factor loading of all the food groups obtained by RRR in women. Table S3. Factor loading of all the food groups obtained by RRR in men. Table S4. Baseline characteristics based on eGFR-based dietary patterns in women. Table S5. Baseline characteristics based on eGFR-based dietary patterns in men. Table S6. Risk of a ≥20% eGFR decline according to baseline Mediterranean Diet Score (0–9) by logistic regression. Table S7. Risk of CKD incidence according to baseline Mediterranean Diet Score (0–9) by logistic regression. Figure S1. Flow chart of participant selection
