# Supplementary material for: Dietary Patterns Based on Estimated Glomerular Filtration Rate and Kidney Function Decline in the General Population: The Lifelines Cohort Study
Source: Nutrients. 2020 Apr 16;12(4):1099. doi: 10.3390/nu12041099 (PMC7230954; doi:10.3390/nu12041099)
Supplement: Supplementary file 1 [file nutrients-12-01099-s001.pdf]

Supplementary Materials

Table S1. Classification of FFQ items in the 50 established food groups in Lifelines cohort

Table S2. Factor loading of all the food groups obtained by RRR in women

Table S3. Factor loading of all the food groups obtained by RRR in men

Table S4. Baseline characteristics based on eGFR-based dietary patterns in women

Table S5. Baseline characteristics based on eGFR-based dietary patterns in men

Table S6. Risk of a ≥20% eGFR decline according to baseline Mediterranean Diet Score (0-9) by logistic regression

Table S7. Risk of CKD incidence according to baseline Mediterranean Diet Score (0-9) by logistic regression

Figure S1. Flow chart of participant selection

Table S1. Classification of FFQ items in the 50 established food groups in Lifelines cohort

Food Groups	FFQ items in Lifelines
Bread and bread products	Rusk, crispbread, crackers, croissants, slices of bread, and other bread
Rice & Pasta	Rice, pasta
Cereals	Muesli, granola or cereals for the preparation of porridges
Potatoes	Boiled or mashed potatoes
Pizza	Pizza
Milk (low/medium fat)	Semi-skimmed/ skimmed milk,regular coffee milk
Milk (full fat)	Full-fat milk, full-fat coffee milk, butter milk,
Yogurt (low fat)	Semi-skimmed plain yogurt
Yogurt (high fat)	Full-fat plain yogurt
Chocolate milk	Chocolate milk
Sweetened dairy drinks	Sugar sweetened milk/yoghurt drinks and other types of diary drinks, skimmed fruit yogurt and quark or fruit quark
Cheese(low fat)	20+ or 30+ cheese or spreadable cheese
Cheese (high fat)	40+ or 48+ cheese or spreadable cheese, cream cheese and/or foreign cheese
Cream products	Whipped cream, coffee cream
Milk-based desserts	Full-fat custard, milk-based ice cream
Eggs	Boiled egg, fried egg
Lean red meat	Beef steak, steak tartare, braising steak or roast beef
Red meat	Sirloin steak, beef bratwurst, beef blade steak, beef rib steak or marbled beef, pork bratwurst, 'slavink' (ground meat wrapped in bacon), pork,
Fatty fish	Salted herring, fried herring, salmon, mackerel, eel and other types of fish
Lean fish	Cod, plaice, haddock, pollack, sole, deep-fried whiting
White meat	Chicken without skin, chicken with skin
Processed meat	Luncheon meats, burger meat, bacon, minced meat (beef or mix of beef and pork), sausage or smoked sausages and other types of meat products
Other meat	Other types of meat or poultry
Nuts and seeds	Peanuts, nuts and seeds, pea-nut butter
Legumes	Brown beans, white beans, marrowfat peas, kidney beans etc.
Soup	Soup with or without legumes
Warm Sauces	Pasta sauce, mushroom sauce, sate sauce

Sauces /dressing/gravy	Regular mayonnaise, low fat mayonnaise, sauce for French fries and other non-red sauces, gravy, salad dressing with/without oil
Edible fat	Butter, margarine, low fat margarine
Commercially prepared dishes	Chinese/Indonesian dishes, meals from fast-food restaurants, other types of ready-to-eat meals
Breakfast drinks	Ready –to-drink breakfast
Fruits	All kinds of fruits
Apple sauce	Apple sauce
Vegetables	Cooked vegetables with/without butter, stir-fried vegetables
Fruit juice	Fruit juice / drink
Fries	Fries and fried potatoes
Cakes and cookies	Small cookies or biscuits, sponge cake, large cookies, cake, pastry, pie
Savory snacks	Croquettes, savory snacks, potato chips or salty biscuits
Sugar and confectionery	Candy bars or candy, chocolate, liquorice, acid drops
Sweet sandwich toppings	chocolate- sprinkles, spread or flakes and other types of sweet fillings
Savory salad topping	Salads or spread on baguette / toast
Wine & fortified wine	Red /rose/white wine, fortified wine (sherry, port, vermouth, madeira)
Beer	Beer
Non-alcoholic beer	Alcohol-free beer
Spirits	Distilled beverages(genever, whisky, rum, gin, cognac, vieux, liqueur) and others
High Sugar beverages	Sugar sweetened beverages such as soft drinks (coke, orange flavored soft drinks, 7-up) or lemonade with sugar
Low Sugar beverages	Soft drinks / lemonade without sugar
Coffee	Coffee with or without sugar and milk, coffee creamer
Tea	Tea with or without sugar
Added sugar	Sugar in yoghurt, thee, coffee

Table S2. Factor loading of all the food groups obtained by RRR in women

Food group	Factor loading
Eggs	0.42
Low-fat cheese	0.23
High-fat cheese	0.23
Legumes	0.20
Savory snacks	0.16
Fatty fish	0.15
Vegetables	0.13
Low Sugar beverages	0.13
Soup	0.11
Bread	0.10
Spirits	0.10
Fruits	0.09
Wine & fortified wine	0.07
Beer	0.06
Full-fat milk	0.04
Sauces dressing gravy	0.04
Non-alcoholic beer	0.04
Low-fat milk	0.03
Lean fish	0.03
Processed meat	0.01
Nuts seeds	0.01
Edible fat	0.01
Breakfast drinking	0.01
Fruits juice	-0.01
Potatoes	-0.01
Chocolate milk	-0.02
Full-fat yogurt	-0.03
Cream	-0.04
Low-fat yogurt	-0.05
Apple sauce	-0.05
Coffee	-0.06
Savory salad topping	-0.07
Tea	-0.07
Pizza	-0.08
Fries	-0.08
High sugar beverages	-0.08
Rice&pasta	-0.09
Warm sauces	-0.10
Added sugar	-0.10
Lean red meat	-0.13
Cereals	-0.16
other meat	-0.16
Red meat	-0.17
Sugar and confectionery	-0.18
Commercially prepared dishes	-0.21
White meat	-0.22
Sweet sandwich toppings	-0.22
Cakes and cookies	-0.24
Dessert	-0.26
Sweetened dairy drinks	-0.27

Table S3. Factor loading of all the food groups obtained by RRR in men

Food group	Factor loading
High-fat cheese	0.38
Bread	0.34
Full-fat milk	0.23
Fruits	0.23
Vegetables	0.21
Beer	0.21
Low-fat cheese	0.20
Legumes	0.20
Potatoes	0.19
Edible fat	0.16
Eggs	0.15
Low-fat yogurt	0.12
Nuts seeds	0.11
Savory salad topping	0.10
Full-fat yogurt	0.09
Apple sauce	0.07
Wine & fortified wine	0.06
Low Sugar beverages	0.06
Chocolate milk	0.05
Warm sauces	0.05
Cakes and cookies	0.05
Coffee	0.05
Fruits juice	0.04
Non-alcoholic beer	0.04
Spirits	0.04
Soup	0.03
Lean fish	0.02
Tea	0.02
Fatty fish	0.01
Savory snacks	0.01
Sweet sandwich toppings	0.01
Cereals	0.01
Dessert	-0.01
Low-fat milk	-0.03
Processed meat	-0.03
Sauces dressing gravy	-0.03
Breakfast drinking	-0.03
Rice&pasta	-0.05
High sugar beverages	-0.05
Added sugar	-0.06
Pizza	-0.07
Sweetened dairy drinks	-0.08
Cream	-0.09
Fries	-0.12
Sugar and confectionery	-0.12
Lean red meat	-0.17
other meat	-0.17
Commercially prepared dishes	-0.17
Red meat	-0.22
White meat	-0.33

Table S4. Baseline characteristics based on eGFR-based dietary patterns in women

	Quartiles of dietary pattern score in women (n = 45,746)			
	**1**	**2**	**3**	**4**
**Demographics**				
Age (year)	41.9±11.8	44.4±12.5	46.3±12.7	48.8±12.1
**Clinical factors**				
Body surface area (m^2)	1.84±0.16	1.84±0.16	1.83±0.16	1.84±0.16
24h urine creatinine clearance (mL/min)	117.7±26.6	116.5±27.2	114.6±28.0	114.5±28.2
Serum creatinine (µmol/L)	67.8±8.8	67.1±8.7	66.7±8.8	65.6±8.9
eGFR (mL/min/1.73 m^2)	96.3±14.4	95.6±14.6	94.9±14.4	94.6±14.0
BMI (kg/m^2)	25.4±4.5	25.7±4.8	25.8±4.5	26.0±4.6
Weight (kg)	73.5±13.5	73.8±13.2	73.6±13.1	74.0±13.6
Waist circumference (cm)	85.6±11.8	86.3±11.7	86.7±11.8	87.4±12.1
Cholesterol (mmol/L)	5.0±1.0	5.0±1.0	5.1±1.0	5.2±1.0
Triglycerides (mmol/L)	0.9±0.5	0.9±0.5	1.0±0.6	1.0±0.6
Diabetes (%)	2.1	2.2	3.2	3.7
Hypertension (%)	19.5	22.2	24.7	26.8
Cardiovascular disease (%)	0.9	1.5	1.8	2.2
**Health-related behaviors**				
Physical activity (minutes/week)	300 (120-644)	360(150-730)	388(150-810)	450(180-900)
Smoker (%)	11.1	13.7	17.1	19.5
Total energy intake (kcal/d)	2076±453	1858±422	1745±433	1738±479
Total protein intake (g/day/1000kcal)	35.8±5.0	37.4±5.1	38.5±5.4	40.3±6.5
Total carbohydrate intake (g/day/1000kcal)	117.5±11.3	114.3±12.0	112.1±13.3	106.5±16.5
Total fat intake (g/day/1000kcal)	39.0±4.8	39.1±5.1	39.0±6.5	39.7±6.5
**Socioeconomic status**				
**Education (%)**				
Low	23.2	27.9	31.6	33.9
Middle	45.4	43.4	39.3	36.5
High	31.0	28.4	28.6	29.1
Unknown/no answer	0.4	0.4	0.5	0.5
**Income (%)**				

Low	8.0	7.2	7.8	7.7
Middle	47.7	48.2	48.8	48.5
High	27.7	28.0	26.5	26.6
Unknown/no answer	16.7	16.6	16.9	17.3

Data are % , means ± sd or median (IQR 25%-75%)

Table S5. Baseline characteristics based on eGFR-based dietary patterns in men

	Quartiles of dietary pattern score in men (*n* =32,589)			
**Characteristics**	**1**	**2**	**3**	**4**
**Demographics**				
Age (y)	44.0±12.4	46.1±12.6	47.4±12.7	48.6±12.6
**Clinical factors**				
Body surface area (m^2)	2.10±0.17	2.10±0.16	2.10±0.16	2.09±0.16
24h urine creatinine clearance (mL/min)	144.1±37.3	142.2±32.4	142.5±32.8	142.3±33.9
Serum creatinine (µmol/L)	83.6±10.4	82.9±10.3	82.1±10.1	80.6±10.1
eGFR (mL/min/1.73 m2)	97.1±14.6	96.4±14.3	96.3±14.0	96.9±13.7
BMI (kg/m^2)	26.6±3.7	26.5±3.6	26.4±3.5	26.0±3.5
Weight (kg)	88.4±13.4	88.2±13.0	88.1±12.7	87.2±12.9
Waist circumference (cm)	95.6±10.7	95.5±10.5	95.3±10.3	94.5±10.5
Cholesterol (mmol/L)	5.1±1.0	5.2±1.0	5.2±1.0	5.2±1.0
Triglycerides (mmol/L)	1.4±1.0	1.4±1.0	1.4±0.9	1.3±0.9
Diabetes (%)	3.3	3.3	4.0	3.7
Hypertension (%)	20.1	19.2	20.9	20.6
Cardiovascular disease (%)	3.9	3.7	4.3	4.5
**Health-related behaviors**				
Physical activity (minutes/week)	365(120-914)	480(165-1087)	600(212-1350)	810(270-1560)
Smoker (%)	19.9	18.0	17.1	18.2

Total energy intake (kcal/d)	2132±567	2260±552	2414±580	2739±694
Total protein intake (g/day/1000kcal)	36.6±5.4	35.9±4.8	35.8±4.7	36.2±5.0
Total carbohydrate intake (g/day/1000kcal)	111.0±14.3	111.9±13.0	111.6±13.2	111.7±13.7
Total fat intake (g/day/1000kcal)	40.2±5.4	39.6±5.1	39.3±5.4	38.8±5.8
**Socioeconomic status**				
**Education (%)**				
Low	25.4	26.6	28.6	31.2
Middle	40.3	39.4	37.1	35.9
High	33.9	33.6	33.8	32.3
Unknown/no answer	0.4	0.4	0.4	0.7
**Income (%)**				
Low	5.2	3.4	2.9	3.5
Middle	49.0	50.7	51.0	52.0
High	34.3	34.6	34.3	31.5
Unknown/no answer	11.5	11.2	11.8	12.9

Data are % , means ± sd or median (IQR 25%-75%)

Table S6. Risk of a ≥20% eGFR decline according to baseline Mediterranean Diet Score (0-9) by logistic regression

**Women**	**Diet Score**			
	**0-3**	**4-5**	**6-9**	**P for trend**
Cases/population	1,734/15,537	2,101/19,633	1,137/10,576	
eGFR decline ≥20%(%)	11.2	10.7	10.8	0.353
Model 1	1.00	0.96 (0.89-1.02)	0.97 (0.89-1.05)	0.420
Model 2	1.00	0.95 (0.89-1.02)	0.96 (0.89-1.04)	0.369
Model 3	1.00	0.95 (0.89-1.02)	0.96 (0.88-1.04)	0.324
Model 4	1.00	0.95 (0.89-1.02)	0.96 (0.89-1.04)	0.376
Model 5	1.00	0.95 (0.89-1.02)	0.95 (0.87-1.03)	0.163

**Men**	**Diet Score**			
	**0-3**	**4-5**	**6-9**	**P for trend**
Cases/population	1,007/12,267	1,139/13,909	492/6,413	
eGFR decline ≥20%(%)	8.2	8.2	7.7	0.382
Model 1	1.00	0.97 (0.88-1.06)	0.88 (0.78-0.99)	0.084
Model 2	1.00	0.96 (0.87-1.05)	0.86 (0.76-0.96)	0.032
Model 3	1.00	0.95 (0.87-1.04)	0.85 (0.76-0.96)	0.027
Model 4	1.00	0.95 (0.87-1.04)	0.86 (0.76-0.96)	0.031
Model 5	1.00	0.95 (0.87-1.04)	0.85 (0.76-0.96)	0.010

Model 1. Adjusted for age and BSA
Model 2. Model 1 plus BMI, waist circumference, cholesterol, triglycerides, diabetes, hypertension and cardiovascular disease
Model 3. Model 2 plus physical activity, smoker, and total energy intake
Model 4. Model 3 plus education and income
Model 5. Model 4 plus baseline eGFR

Table S7. Risk of CKD incidence according to baseline Mediterranean Diet Score (0-9) by logistic regression

Women	Mediterranean Diet Score			
	**0-3**	**4-5**	**6-9**	**P for trend**
Cases/population	335/15,537	584/19,633	343/10,576	
CKD incidence (%)	2.2	3.0	3.2	<0.001
Model 1	1.00	0.93 (0.80-1.07)	0.83 (0.70-0.97)	0.060
Model 2	1.00	0.92 (0.80-1.06)	0.82 (0.70-0.97)	0.060
Model 3	1.00	0.92 (0.80-1.06)	0.82 (0.70-0.97)	0.056
Model 4	1.00	0.93 (0.80-1.07)	0.82 (0.70-0.97)	0.062
Model 5	1.00	0.92 (0.78-1.08)	0.88 (0.73-1.06)	0.174

Men	Mediterranean Diet Score			
	**0-3**	**4-5**	**6-9**	**P for trend**
Cases/population	241/12,267	353/13,909	216/6,413	
CKD incidence (%)	2.0	2.5	3.4	<0.001
Model 1	1.00	0.80 (0.67-0.95)	0.76 (0.62-0.93)	0.013
Model 2	1.00	0.80 (0.68-0.96)	0.76 (0.62-0.94)	0.015
Model 3	1.00	0.80 (0.66-0.94)	0.74 (0.61-0.91)	0.007
Model 4	1.00	0.79 (0.66-0.94)	0.74 (0.60-0.91)	0.007
Model 5	1.00	0.86 (0.70-1.05)	0.87 (0.69-1.10)	0.243

Model 1. Adjusted for age and BSA

Model 2. Model 1 plus BMI, waist circumference, cholesterol, triglycerides, diabetes, hypertension and cardiovascular disease

Model 3. Model 2 plus physical activity, smoker, and total energy intake

Model 4. Model 3 plus education and income

Model 5. Model 4 plus baseline eGFR

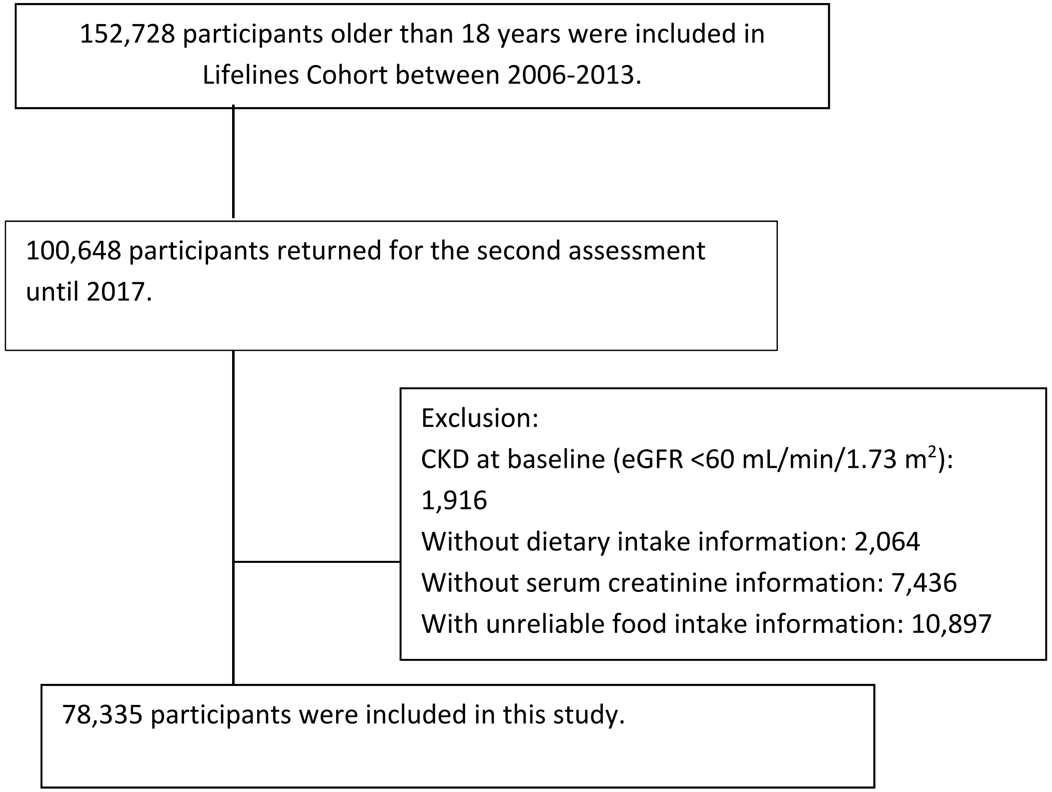

Figure S1. Flow chart of participant selection
